# Cow’s Milk Allergy in Breastfed Infants: What We Need to Know About Mechanisms, Management, and Maternal Role

**DOI:** 10.3390/nu17111787

**Published:** 2025-05-24

**Authors:** Carlo Caffarelli, Arianna Giannetti, Enrico Vito Buono, Daniela Cunico, Roberta Carbone, Federica Tonello, Giampaolo Ricci

**Affiliations:** 1 Clinica Pediatrica, Dipartimento di Medicina e Chirurgia, University of Parma, 43126 Parma, Italy; enricovito.buono@unipr.it (E.V.B.); daniela.cunico@unipr.it (D.C.); roberta.carbone2@unipr.it (R.C.); 2 Paediatrics Unit, IRCCS Azienda Ospedaliero-Universitaria di Bologna, 40138 Bologna, Italy; arianna.giannetti@aosp.bo.it; 3 Specialty School of Pediatrics, Alma Mater Studiorum, University of Bologna, 40138 Bologna, Italy; federica.tonello@studio.unibo.it; 4 Department of Medical and Surgical Sciences (DIMEC), University of Bologna, 40138 Bologna, Italy; giampaolo.ricci@unibo.it

**Keywords:** cow’s milk allergy, breastfed infants, IgE-mediated reactions, non-IgE mediated reactions, diagnosis, microbiota

## Abstract

Cow’s milk allergy is one of the most prevalent food allergies in infancy. Exclusive breastfeeding is the recommended source of nutrition for the first six months of life, but some infants may develop cow’s milk allergy due to the transfer of milk proteins such as β-lactoglobulin through breast milk. There are still many uncertainties about cow’s milk allergy in breastfed babies. The purpose of this review is to summarize the latest findings mainly focused on immunological mechanisms and challenges in diagnosis. We pointed out which clinical signs in breastfed infants are associated with immediate IgE responses and which are linked to delayed non-IgE mechanisms or mixed IgE/non-IgE-mediated reactions. Since standard IgE tests are often useless in non-IgE cases, diagnosis typically involves dietary elimination and cow’s milk challenge. This study addresses the controversial topic of maternal elimination diets, assessing the nutritional risks to both mothers and infants in relation to their possible benefits. It has also been discussed whether the microbiota signature could be a potential factor in both tolerance development and the risk of cow’s milk allergy in early life. Overall, accurate diagnosis and personalized treatment plans are vital to prevent overdiagnosis and ensure proper growth while maintaining the practice of breastfeeding.

## 1. Introduction

Milk is the initial food given to infants, no matter where they live or their cultural background, and it plays a key role in their diet. Cow’s milk allergy (CMA) is an immune-mediated reaction to proteins of cow’s milk, including casein, α-lactalbumin, and β-lactoglobulin (β-LG), which happens regularly after consumption. Based on the underlying immune mechanisms, CMA can be classified into IgE-mediated CMA, non-IgE-mediated (cell-mediated) CMA, or a mixed form involving both IgE and cell-mediated responses. Cow’s milk is among the most common cause of food allergy in infants. [1]. The prevalence of CMA ranges from less than 1% to as high as 7.5% in infants younger than 1 year. Data heterogeneity reflects the influence of several factors such as age, breastfeeding history, diagnostic criteria, and geographic region [2]. Most children with CMA become tolerant by the age of 5, and the rates of tolerance continue to rise throughout childhood and adolescence [3,4,5,6]. Oral tolerance to food antigens takes place when food proteins cross the mucosal barrier, are processed by dendritic cells in a “non-activated state”, and induce the production of suppressive cytokines, such as interleukin-10 (IL-10). This leads to the differentiation of naïve T cells into regulatory T cells (Tregs) and the suppression of food antigen-specific Th2 cells. Moreover, this mechanism increases IgA and IgG4 production and decreases IgE levels. The immune suppression of eosinophils, basophils, and mast cells further contributes to preventing allergic reactions [7,8]. Exclusive breastfeeding is the recommended nutritional source for the first six months of life. Nevertheless, it is well known that exclusively breastfed infants can develop allergic reactions to certain foods, most notably cow’s milk, egg, peanut, and fish [1]. There are very few studies on the frequency of CMA in breastfed babies. In a birth cohort of 1749 infants, the incidence of CMA confirmed by positive milk challenge was 2.2% in the first year of life and 0.5% during exclusive breastfeeding [9]. In a prospective study in 751 newborns, the overall incidence of CMA was 1.9% within the first year of age, and 0.5% of babies had CMA when exclusively receiving breast milk [10]. Studies show that ingested allergenic proteins such as ovalbumin, β-lactoglobulin, gliadin, and peanut proteins (Ara h2 and Ara h6) can pass into human breast milk at low levels, but their clinical significance remains uncertain [1,11,12,13,14,15]. The link between the presence of allergens in maternal milk and CMA is still unclear [16,17,18,19,20,21,22,23,24]. Additionally, diagnosis can be challenging because the definitive method relies on the resolution of symptoms when the nursing mother follows a milk-free diet and the reappearance of symptoms upon reintroducing milk into the diet. A thoughtful reflection is essential before starting a maternal avoidance of cow’s milk as it can impair the intake of essential nutrients [25,26]. The reluctance to recommend a maternal diet is supported by a web-based survey showing that nursing mothers with infants who had IgE-mediated food allergy to allergens contained in breast milk were advised by a healthcare provider to continue the intake of the offending food in 43% of cases and to suspend intake in 17% of cases, and in others, the concern was not tackled [27]. Moreover, it should be considered that an inadequate diagnostic work-up results in the overdiagnosis of CMA, which can negatively impact growth, family dynamics, and socioeconomic status [28]. This review seeks to enhance our understanding of the role of breastfeeding in triggering clinical allergic reactions to cow’s milk, as well as the diagnostic procedures required to identify CMA in breastfed infants. We conducted a literature search using the PubMed database to collect information, using keywords such as “cow’s milk allergy”, “breastfeeding”, “elimination diet”, “microbiota”, and “children” to retrieve articles. Articles published in the past 10 years up to December 2024 and written in English were searched. We only considered articles that were significant for the purpose of this review. In addition, relevant articles that the authors knew or recognized in the references of the selected articles were considered.

## 2. Cow’s Milk Allergens in Breast Milk and Risk of Developing Reactions

Cow’s milk contains about 30–35 g of proteins per litre, with roughly 80% being caseins and the remaining 20% being whey proteins. β-lactoglobulin (β-LG) is a whey protein that is found in the milk of cows, goats, and sheep but not in human milk. β-LG is closely linked to cow’s milk allergy, though caseins and other milk proteins can also potentially trigger allergic reactions. β-LG constitutes a globular protein that forms a dimer composed of 18 kDa monomer units and demonstrates strong resistance to acid and enzymatic digestion in humans [29]. Caseins occur in greater amounts than whey proteins in bovine milk. However, they are broken down quickly in the stomach and subsequently in the intestine [30]. β-LG is the most frequently detected cow’s milk protein in human milk in clinical studies, while casein was measured only once [15]. In breast milk, β-LG levels usually range from 0 μg/L (in 42–52% of studies) to 8 μg/L and can reach up to 800 μg/L, while in bovine milk, the content is 3.3 g/L. [15,31,32,33,34]. Notably, variations in β-LG levels were observed even among women within the same study despite consuming the same amount of cow’s milk. These differences are likely influenced by physiological variations in the absorption of food proteins in the maternal gut and their subsequent transfer into breast milk [15]. Other authors have emphasized that high β-LG levels in breast milk are associated with prolonged cow’s milk intake [35,36,37]. Measurable concentrations of β-LG were detected up to 7 days after maternal intake of cow’s milk [38]. Studies aimed at estimating the peak allergen levels following maternal ingestion of cow’s milk have shown that the highest concentrations occur between 4 and 6 h after consumption [39,40]. It is noteworthy that it has been demonstrated that the detection of β-LG in the breast milk of exclusively breastfed infants with atopic dermatitis is associated with the measurement of β-LG in infant sera. This provides evidence that β-LG contained in breast milk is transferred into the circulation of infants after maternal breast milk intake [23]. When examining the stage of lactation and comparing allergen concentrations in colostrum, transition milk, and mature milk, no higher levels of allergens were found in colostrum compared to transition milk [32], nor in transition milk compared to mature milk [41]. Two studies compared colostrum from mothers of preterm and term infants. In these studies, β-LG was not detected in either preterm or term human colostrum. However, bovine alpha-S1-casein was found in human colostrum, with higher concentrations of S1-casein in the colostrum of mothers with preterm infants [42,43]. Several studies have investigated the concentration of allergens in breast milk with respect to the mother’s allergy status. Some researchers found no correlation between maternal allergies and β-LG levels in breast milk [32,38,39], while others observed elevated β-LG levels in mothers with atopic conditions [33,44,45]. Additionally, some studies highlighted significant variations in protein levels between the breast milk of allergic and non-allergic mothers. Allergic mothers had much higher concentrations of protease inhibitors and apolipoproteins in their milk. Previous studies have proposed a potential link between these proteins and the development of allergies and asthma [45]. A significant disparity in non-human protein levels has been detected in the breast milk of allergic and non-allergic mothers. This difference is mainly attributed to sequences that match with bovine proteins like β-LG and 2-HS-glycoprotein [46]. β-LG levels in the breast milk of mothers with allergic children were compared to those in the milk of mothers with healthy infants in two studies, which found no correlation between the child’s allergic condition and the presence of cow’s milk allergens in the milk [9,47]. Overall, data on allergenic food protein levels in breast milk following maternal ingestion may lead to the estimation that more than 99% of infants with CMA may tolerate breastmilk from a mother who consumes dairy products [12]. Gamirova et al. [15] estimated that the likelihood of developing an allergic reaction following the meal was 1 in 2893 breastfed infants. So, maternal allergen avoidance could be a possible primary approach in the management of CMA [1,48], but dietary restrictions in a breastfeeding mother are often unnecessary [13,14]. Some studies have identified IgE cross-reactivity between cow’s milk proteins and human milk proteins, such as α-lactalbumin, in individuals with CMA [37,49,50]. Additionally, infants showed symptoms even when their mothers adhered to a strict cow’s milk-free diet [1]. This may suggest that symptoms may persist in exclusively breastfed infants with CMA, even when the mother is on a diet free of cow’s milk proteins. Breastfeeding is linked to exposure to aero allergens, too. A French cohort study by Baïz et al. [51] detected Der p 1 in two-thirds of human milk samples, with higher levels found in atopic mothers. Elevated exposure to Der p 1 in breastfed infants of atopic mothers was associated with increased risk of asthma, allergic rhinitis, and higher IgE levels by the age of five. These findings suggest that early oral exposure to certain antigens may contribute to allergic sensitization in children, underscoring the importance of carefully designing studies on early antigen exposure for allergy prevention.

This is accompanied by the fact that breast milk can contain allergenic food proteins, so it is not surprising that breastfed infants develop clinical conditions related to the foods consumed by their mothers.

## 3. Conditions Related to CMA

Phenotypes of CMA depend on the underlying mechanisms involved. Approximately 60% of individuals have IgE-mediated CMA, a type I hypersensitivity reaction, with symptoms occurring within minutes to 1–2 h after milk ingestion. Non-IgE-mediated and mixed mechanisms of CMA also exist.

### 3.1. IgE-Mediated Reactions

When a food antigen crosses a damaged epithelial barrier, it triggers the release of danger signals and inflammatory cytokines; this causes dendritic cells to go into an activated defence state, mimicking a response to pathogens, resulting in the activation of T helper cell 2 (Th2) responses, which promote inflammatory signalling and induce food antigen-specific B cells to produce IgE antibodies. IgE-mediated reactions are type I hypersensitivity reactions that work when two adjacent IgE molecules bind to a food protein. This linking causes the degranulation of mast cells and basophils, leading to the rapid release of preformed mediators. Symptoms appear a few minutes after ingestion; in infants, they usually occur immediately after breastfeeding. IgE-mediated CMA is rare in exclusively breastfed infants, but some case reports have documented allergic reactions due to cow’s milk protein transfer through human milk [7]. The symptoms of IgE-mediated CMA are depicted in Table 1 [11,16,52].

Järvinen et al. [40] investigated the effects of maternal cow’s milk consumption on breastfed infants with CMA through a controlled cow’s milk challenge. Seventeen mothers with CMA-diagnosed infants and ten mothers with healthy infants participated. Among the mothers of CMA infants, 65% were atopic compared to 50% of mothers with healthy infants. The geometric mean age of infants with CMA was 5.4 months compared to 5.1 months in the healthy group. CMA symptoms typically began at a mean age of 1.7 months, with 14 of 17 affected infants being exclusively breastfed at symptom onset. In contrast, most healthy infants were exclusively breastfed until 4–5 months, except for three who received cow’s milk formula early in life. During the cow’s milk challenge, all but one infant with CMA reacted to cow’s milk proteins transferred through human milk. One infant displayed immediate urticaria upon skin contact with cow’s milk but proceeded with the oral challenge. Nine infants with mild reactions to maternal milk underwent further oral cow’s milk provocation to confirm the diagnosis. Notably, the single infant who did not react through breastfeeding responded positively to direct oral cow’s milk exposure. Cutaneous reactions were universal among CMA infants, with five experiencing gastrointestinal symptoms and three showing upper respiratory issues (e.g., rhinorrhea and acute otitis media). The median maternal cow’s milk intake that elicited infant symptoms was 700 mL (range: 100–2300 mL), with reactions typically appearing within 21 h (range: 2–80 h). In contrast, all control infants exhibited negative responses to both breastfeeding and direct cow’s milk exposure. Skin prick tests for cow’s milk were negative in all but 1 CMA infant, though 3 of 17 had positive reactions to egg. No infants demonstrated sensitivity to their mother’s milk via skin prick test. Anaphylactic reactions to human milk are rarely reported, and while no prevalence estimates exist, they are considered an uncommon manifestation of food allergy in exclusively breastfed infants. Recognition of anaphylaxis in this population can be challenging, as symptoms often present differently and may be nonspecific [53]. Lifschitz et al. [54] described a 5-day-old breastfed infant who developed proctitis with visibly bloody stools. On day 8 of life, the infant was switched to a casein-based formula. After the third feeding, the infant became lethargic and experienced profuse vomiting, bloody loose stools, hypotension, and cyanosis, though the timing of the reaction was not specified. In response, the mother eliminated all CM from her diet. At 3 weeks of age, the infant resumed breastfeeding without symptoms. However, accidental ingestion of previously frozen breast milk, expressed before maternal CM elimination, triggered cyanosis, undetectable blood pressure, and mucus bloody stools and necessitated resuscitation. Although initially tolerating a casein hydrolysate formula, the infant experienced another reaction after two days, presenting with duskiness, floppiness, hypotension, and facial swelling, which were managed with epinephrine. A laboratory evaluation revealed elevated peripheral blood eosinophils and positive specific IgE to alpha-lactalbumin and β-lactoglobulin. Despite these severe reactions, the infant outgrew the allergy and tolerated direct ingestion of cow’s milk by 12 months of age. Two cases of immediate allergic reactions to human milk following maternal fish consumption have been documented in exclusively breastfed infants [55,56]. These rare case reports highlight that food proteins from a maternal diet can elicit anaphylactic symptoms in breastfed infants, underscoring the importance of early recognition and appropriate management of food-induced reactions in this vulnerable population.

### 3.2. Non-IgE-Mediated and Mixed IgE/Non-IgE-Mediated Reactions

In non-IgE-mediated CMA, the symptoms are often quite nonspecific and can overlap with functional conditions related to gut–brain interaction. Manifestations mainly include gastrointestinal symptoms (vomiting, diarrhea, and sometimes malabsorption) due to gastrointestinal inflammation and dysmotility. Society guidelines [14,28,57] often offer inconsistent approaches to these disorders.

#### 3.2.1. Food Protein-Induced Proctocolitis

Food protein-induced proctocolitis (FPIP), also known as allergic colitis, eosinophilic proctocolitis, or food protein-induced allergic proctocolitis, is determined by immune-mediated reactions to one or more allergens that cause inflammatory changes in the distal colon and rectum. The signs and symptoms generally appear during the first months of life, and most cases resolve by 1 year of age. FPIP usually presents with bloody stools with or without mucus or diarrhea. Infants are generally in good health, showing regular growth, and weight loss or anatomical anomalies are typically not reported. Some of them are fussy and irritable. FPIP is diagnosed clinically as there is currently no diagnostic test available. Exclusively breastfed infants are involved in 56–68% of FPIP cases [58,59]. Cow’s milk proteins are the primary cause of FPIP, responsible for up to 65% of cases. Soy, egg, and wheat are also trigger foods [60]. In a prospective cohort study involving 13,019 newborns, the frequency of FPIP due to cow’s milk proteins was found to be 1.6 per 1000 infants. The diagnosis was confirmed by an oral food challenge after an elimination diet [61]. FPIP occurs due to maternal ingestion of cow’s milk in 18% of breastfed infants [17]. The diagnosis is based on the efficacy of maternal diet without cow’s milk proteins. Rectal bleeding typically resolves macroscopically within 72–96 h from the beginning of the diet, with the complete resolution of symptoms within 1–2 weeks. The reintroduction of cow’s milk proteins following a period of a therapeutic elimination diet should be carried out at home after 6 months of the diet or after 1 year of age. IgE tests and recto-sigmoidoscopy are not generally necessary. The skin prick test for cow’s milk proteins usually yields negative results, but it can become positive over time. In such cases, cow’s milk should be reintroduced under medical supervision. Recto-sigmoidoscopy with rectal biopsy should be limited to cases that do not improve with restricted diet and when other diagnoses need to be considered. It shows mucosal congestion with petechial areas. Inflammation is characterized by eosinophilic infiltration and lymph nodular hyperplasia [62]. Taking into account that the disease is benign and self-resolving, there are conflicting opinions on the appropriateness of starting a dairy-free diet in nursing mothers. In selected cases of long-lasting and severe haematochezia, or in response to concerns raised by parents, it may be advisable to consider eliminating cow’s milk from the maternal diet.

#### 3.2.2. Food Protein-Induced Enterocolitis Syndrome

Food protein-induced enterocolitis syndrome (FPIES) symptoms can vary in severity depending on the frequency and amount of triggering food consumed. The onset of FPIES following maternal intake of cow’s milk is uncommon, and it is only described in case reports [18,19,20,21,22,63,64]. Acute FPIES is triggered by accidental maternal ingestion of dairy products. Acute FPIES usually manifests with repeated prolonged vomiting, lethargy, and pallor occurring approximately 1 to 4 h after consuming the offending food. Watery diarrhea (occasionally with blood and mucous) develops in some cases within 4–8 h of ingestion and can be present for up to 24 h. Most infants with acute FPIES are healthy and exhibit normal growth. In breastfed infants, FPIES caused by cow’s milk is often a chronic condition characterized by intermittent vomiting that is not clearly linked to meals. Symptoms can also include diarrhea (sometimes with blood), dehydration, metabolic acidosis, neutrophilia, thrombocytosis, and failure to thrive. The appearance is similar to what is observed during sepsis. FPIES to cow’s milk generally resolves within 3 years of age. The tolerance achievement is ascertained by oral food challenge that is usually performed 12–18 months following the last reaction [65].

#### 3.2.3. Other Non-IgE-Mediated Conditions

There are some case reports of food protein-induced enteropathy (FPE) in breastfed infants that were triggered by cow’s milk [66] or egg [67]. FPE is characterized by chronic diarrhea that can lead to malabsorption with hypoalbuminemia and swelling at the extremities, weight loss, and failure to thrive. Mucus, abdominal bloating, and recurrent vomiting may be present [68]. Jejunal biopsy is required for diagnosing FPE. Histological findings include mild villous atrophy, crypt hyperplasia, and eosinophilic infiltration. The resolution of FPE typically occurs before 3 years of age [68].

It has been found that in breastfed infants with gastroesophageal reflux disease, a cow’s milk elimination diet lasting 10 to 14 days can improve symptoms, decrease acid reflux, and enhance esophageal mucosal impedance. Symptoms recurred after a 3–7-day oral challenge with cow’s milk [69]. It has been suggested that in infants <1 year of age with proven gastroesophageal reflux disease, a trial consisting of a cow’s milk elimination diet should be considered when standard treatment, including smaller feeds, thickening of feeds, positioning, and avoidance of overfeeding, is unsuccessful [70]. A 2–4-week maternal cow’s milk-free diet followed by a positive 1–2-week challenge would be needed for diagnosis [70]. It should always be considered that gastroesophageal reflux is transitory, and therefore, improvement may not be linked with diet.

It appears that other non-IgE-mediated reactions to cow’s milk, including Heiner syndrome (pulmonary hemosiderosis) and constipation [71], have not been properly investigated in breastfed infants.

### 3.3. Mixed IgE/Non-IgE-Mediated Reactions

Eosinophilic esophagitis has been reported to be diagnosed before the age of 1 year in 3% of 301 patients with documented eosinophilic esophagitis who were younger than 18 years old. However, it was not specified how many of these infants were breastfed [72]. It is unclear whether eosinophilic esophagitis, eosinophilic gastroenteritis, and eosinophilic colitis due to CMA can develop during breastfeeding.

#### Eczema/Atopic Dermatitis

Eczema/atopic dermatitis (AD) is considered a T2 chronic inflammatory disease. The underlying mechanism remains debated. It involves a complicated interaction between a compromised skin barrier, pruritus, skin inflammation, and immune dysregulation, all influenced by both genetic and environmental factors. The onset of AD occurs early in life, with a prevalence of approximately 20% and an incidence of 9.6% among infants and children in Westernized countries. The presence of a family history of atopy or AD has been well recognized as a risk factor for CMA. Some studies suggest that AD may be a possible mixed form of CMA in some young children, including breastfed infants. CMA occurs alongside early and severe AD in a significant number of infants. CMA can cause eczema flare-ups that usually happen between 6 and 48 h after breastfeeding. In a Danish cohort of 1749 infants, 39 (2.2%) developed CMA. Among them, nine were exclusively breastfed; of these, eight had AD with or without diarrhea, vomiting, rhinitis, urticaria, and recurrent wheezing, which responded to maternal dairy-free diet. Identical symptoms recurred when the mother ingested a minimum of 0.5 L of cow’s milk daily [9]. In another study, 4 out of 20 Swedish children with CMA diagnosed in their first year of life presented gastrointestinal and cutaneous symptoms (colic, vomiting, diarrhea, rash, and AD) during exclusive breastfeeding. The manifestations disappeared with a maternal cow’s milk elimination diet and reappeared following the reintroduction of milk in the maternal diet [10].

It has been found [23] that AD improves following maternal avoidance of cow’s milk in breastfed babies with and without serum IgE to cow’s milk. AD resolution was associated with a lack of β-LG in maternal milk and in the circulation of the infant, while skin symptoms relapsed following maternal ingestion of cow’s milk that was associated with the detection of β-LG in both maternal milk and infants’ sera [23]. It has also been reported that in 16% of breastfed babies, AD improved after the mother eliminated eggs and cow’s milk from her diet, but symptoms worsened when these foods were reintroduced [24].

In summary, CMA presents with diverse phenotypes. IgE-mediated reactions cause a quick onset of hypersensitivity symptoms, whereas non-IgE-mediated and mixed forms primarily result in gastrointestinal inflammation and other chronic symptoms like FPIP, FPIES, FPE, and AD, often influenced by maternal diet and typically resolving within the early years of life.

## 4. Diagnostic Issues

The diagnosis of CMA in breastfed babies embraces detailed history, allergy tests (skin testing and/or specific IgE antibodies to milk proteins), and oral food challenge (OFC) that is considered the gold standard for diagnosis. IgE testing is not typically suggested in diseases not mediated by IgE except if the child presents with immediate-type symptoms or during follow-up to assess for the development of IgE sensitization. The diagnosis of CMA in exclusively breastfed infants may be challenging. For example, symptoms associated with suspected allergy, such as occasional traces of blood, are common neonatal conditions and are therefore not sufficient for a definitive diagnosis. Symptoms that are suspected to be due to CMA must be carefully defined, and their resolution after milk elimination must be objectively assessed [8]. While documented cases of food allergy in breastfed infants exist, they are rare, and efforts should be made to prevent overdiagnosis. Ingested allergens, including cow’s milk proteins, may reach the infant through breast milk, but the quantities are typically small and often not clinically relevant. Guidelines provide various diagnostic approaches [28,62,65,68,73,74]. Guidelines suggest continuing breastfeeding and eliminating suspected allergens from the maternal diet when the infant has clinical symptoms. However, strict maternal elimination diets can be challenging to maintain and may lead to nutritional deficiencies. Additionally, evidence supporting maternal elimination diets to manage food allergy in breastfed infants is limited due to a lack of high-quality randomized controlled trials and variability in allergen secretion into human milk [28]. We suggest a diagnostic strategy that varies depending on whether the suspected allergy is mediated by IgE or not as indicated by the results of skin prick tests and IgE-specific antibodies to cow’s milk. The duration of the diagnostic maternal elimination diet should be based on the clinical condition. Maternal dietary restrictions should be reserved for persistent infant symptoms without alternative explanations [28]. Regularly avoiding foods with precautionary allergen labels is not recommended [65]. In IgE-mediated CMA, a maternal diet consisting of the avoidance of cow’s milk proteins should be followed for 2–3 days (Table 2). In non-IgE-mediated or mixed CMA, the length of the maternal diet should be 48–72 h in FPIP, 2 days in acute FPIES and 3–7 days in chronic FPIES, 1–4 weeks in FPE, and 15 days in AD. The persistence of symptoms despite a maternal diet free of cow’s milk requires the assessment of an alternative diagnosis. Reintroduction is recommended for diagnosis, except in cases of severe symptoms or confirmed FPIES [74]. In infants with non-IgE-mediated (with the exception of FPIES) or mixed CMA, the mother should gradually introduce milk in their diet at home if the results of a skin test to cow’s milk are negative. Oral food challenge can be considered in order to confirm the diagnosis of FPIES, especially in cases of chronic FPIES or when there are less than two episodes or if an episode did not require a visit to the emergency department. In other conditions, it is recommended that the oral provocation test is performed under the surveillance of a physician. If the symptoms resolve, the suspected food should be reintroduced into the mother’s diet. If the symptoms return, this confirms the diagnosis of CMA. Otherwise, the mother can resume a normal diet.

Overall, the diagnosis of CMA in breastfed infants requires careful symptom evaluation, targeted allergy testing, a maternal elimination diet of variable duration depending on whether the allergy is IgE- or non-IgE-mediated, and confirmation through reintroduction or oral food challenge while avoiding unnecessary diagnoses and dietary restrictions.

## 5. Nutritional Considerations

The nutritional composition of breast milk is dynamic and influenced by numerous factors, including the mother’s health and dietary habits. While a mother’s diet does not need to be flawless to adequately nourish the baby, it does play a role in determining the nutrients present in the milk. Eliminating nutritionally important foods such as dairy, eggs, wheat, soy, fish, and nuts—particularly when multiple foods are removed—requires careful dietary planning to maintain the nutritional quality of breast milk. Certain nutrients, like calcium, iron, zinc, copper, and folate, are relatively unaffected by maternal diet. In contrast, nutrients such as thiamine, riboflavin, vitamins B6 and B12, choline, vitamins A and D, selenium, iodine, and key fatty acids like docosahexaenoic acid (DHA) are more directly influenced by what the mother consumes, making it important to monitor and support maternal nutrition during breastfeeding [75,76,77]. In cases of extended maternal elimination diets, supplementation with calcium and vitamin D is recommended, while iodine and vitamin B12 supplementation may be considered [78,79,80]. A study by Adams et al. [25] found that breastfeeding mothers following cow’s milk and dairy-free diets exhibited increased bone turnover despite receiving adequate calcium supplementation of 1000 mg/day. These mothers also had lower intakes of energy, protein, and phosphorus compared to those on unrestricted diets. One additional drawback of eliminating dairy products during lactation is the avoidance of yoghurt and other fermented dairy foods, which are primary sources of probiotics for many women. Although probiotic supplements are available, fermented dairy products offer extra benefits for maternal gut health. The bacterial enzymes in these foods convert lactose into lactic acid and transform milk carbohydrates into oligosaccharides with prebiotic properties, supporting a healthier gut microbiome [81]. Given the growing body of evidence highlighting the importance of a balanced gut microbiome for overall health, the potential loss of these benefits is a significant consideration when recommending maternal cow’s milk elimination. Professional dietary counselling is advised to ensure the mother’s diet remains nutritionally adequate [28].

Maternal elimination diets during breastfeeding, especially when excluding multiple nutrient-rich foods like dairy, require careful planning and professional guidance to maintain breast milk quality and maternal health as they can impact key nutrient levels, bone health, and gut microbiota balance.

## 6. Tolerance Development

In most cases, infants with CMA achieve tolerance before adulthood. So far, there are no biomarkers, except for patients with positive skin prick test results who exhibit negative skin tests in the follow-up [82], that can identify children with allergies who have become tolerant to milk. Therefore, the timing of reintroducing dairy products in lactating mothers after CMA diagnosis has not been well defined. It is unclear whether tolerance to cow’s milk will be faster in IgE-mediated than in non-IgE-mediated allergy. According to some suggestions, a food elimination diet can last from 3 months to 6 months for non-IgE-mediated CMA [62,83]. However, the development of tolerance can also occur over time, lasting up to adolescence, in IgE-mediated CMA. In any case, an effort should be made to limit the duration of the elimination diet with periodic milk reintroduction attempts.

## 7. The Microbiota: Navigating Between Prevention and Treatment

Human milk is a vital source of microbes, containing between 10^7^ and 10^8^ bacterial cells per 800 mL of milk [84]. These microorganisms, which are not just contaminants from the skin, colonize the infant’s gut lumen [85]. The microbiota of the birth canal in vaginal delivery are another source from which infants acquire gut microbiome. Variations in the composition of the intestinal microbiota is associated with the development of allergic diseases, including food allergy and asthma [86]. For example, it has been demonstrated that the combination of the breast milk probiotics L. gasseri CECT5714 and L. coryniformis CECT5711 reduces both the incidence and severity of allergic reactions in an animal model of CMA [87]. Wang et al. [88] conducted a study in a small cohort of mother–infant pairs. Babies were exclusively breastfed until 6 months of age. The pairs were categorized based on whether the infant had a food allergy by the age of 1 year. Samples of intestinal microbiota of exclusively breastfed infants at 1 day, 3 days, 1 month, 3 months, and 6 months after birth were collected and analyzed. In agreement with a previous study [89], they found that the relative abundances of *Proteobacteria*, *Acinetobacter*, and *Pseudomonas* in breast milk microbiota was significantly higher in the food allergy group, while there were higher abundances of *Akkermansia*, *Bifidobacterium*, *Clostridium IV*, *ClostridiumXIVa*, and *Veillonella* in the non-allergy group. *Prevotella* was also higher in the breast milk microbiota of the non-allergy group than in the allergy group. However, there was no difference in the relative abundance of *Prevotella* in the intestinal microbiota of infants between the two groups, suggesting that it does not influence food allergies by changing intestinal microbiota. This study also showed relative abundances of some butyrate-producing bacteria, such as *Fusobacterium*, *Lachnospiraceae incertae sedis*, *Roseburia*, and *Ruminococcus*, in the non-allergy group compared to the allergy group. This finding agrees with previous studies that proposed that butyrate in breastmilk could prevent the onset of food allergies [90]. In fact, butyrate suppresses the production of pro-inflammatory cytokines and enhances anti-inflammatory activity. This phenomenon is illustrated by the fact that children with CMA typically have lower levels of butyrate at one year of age [91]. In general, children with CMA tend to exhibit reduced microbiome diversity, which may lead to a dysbiotic imbalance, higher intestinal permeability [92], and compromised membrane integrity [93]. In particular, an elevated ratio of Enterobacteriaceae (Proteobacteria and Actinobacteria) and Bacterioidaceae (Firmicutes and Bacteroidetes), known as the E/B ratio, was found in the gut microbiota of infants allergic to cow’s milk at the time of diagnosis (abundance of Ruminococcaceae and Lachnospiraceae) when compared to age-matched healthy 4-month-old controls (dominated by Bifidobacteriaceae, Enterobactericeae, and Enterococceae) [94]. These modifications seem to be crucial in the development of the disease and the appearance of symptoms. A Th2-dominant response that can promote allergic reactions is associated with disruptions in the gut flora. A balanced gut microbiota helps maintain immune equilibrium between Th1 and Th2 cells. However, autoimmune disorders that are associated with a Th1 immune response and allergic conditions may coexist within a single individual [95]. *Bifidobacterium* is one of the predominant microbes in breastfed infants, and its levels are higher in breastfed babies compared to those who are formula-fed. This bacterium is associated with increased levels of Treg cytokines such as IL-27, IL-10, and IL-6 [96]. Additionally, *Bifidobacterium* appears to reduce inflammation and help restore immune balance early in life by decreasing Th2 and Th17 cytokines in the intestines while promoting the production of IFN-γ. Prebiotic (galacto-oligosaccharides/inulin) in mice increased the relative abundances of *Lactobacillus* and *Bifidobacterium*. Furthermore, it has been demonstrated that the oral administration of galacto-oligosaccharides/inulin during pregnancy and lactation reduced TGF-β1 and TGF-β2 levels and allergy symptoms related to wheat allergy [97]. Biotics are bioactive substances that exert nutritional effects. This category includes probiotics, which are live microorganisms. Among these, *Lactobacillus rhamnosus GG (LGG)* is one of the most extensively studied strains. Cox also investigated how the gut microbiome changes when LGG is added to infants’ diets, discovering that higher doses of LGG resulted in alterations to the gut microbiota [98]. However, it is still uncertain whether adding *LGG* to an extensively hydrolyzed formula can reduce the development of other allergic diseases in children with CMA [3,99]. Furthermore, adding *LGG* to infant formula does not aid in the acquisition of tolerance to cow’s milk among infants with both IgE- and non-IgE-mediated CMA. Even though many studies have found that microbiota transmitted through breastfeeding can be protective against food allergies, the current data do not definitively recommended probiotics as a preventive measure for CMA.

## 8. Perspectives for Future Research

While progress has been made in understanding how the maternal diet affects food allergies in breastfed infants, significant knowledge gaps remain. Future research should prioritize clinical applications that improve diagnosis, treatment, and long-term outcomes. Future research should focus on improving diagnostic methods through high-quality randomized controlled trials to standardize the diagnosis of CMA in exclusively breastfed infants. These studies should incorporate objective criteria and explore non-invasive diagnostic tools, including the development and validation of reliable biomarkers for non-IgE-mediated allergies, which often present with ambiguous gastrointestinal symptoms and are currently difficult to confirm. Investigating how allergens are transferred through breast milk, influenced by factors such as maternal gut permeability, genetics, and microbiome composition, could lead to personalized management strategies. Research should determine whether shorter elimination periods are as effective as prolonged ones, particularly for conditions like FPIES and FPIP. Additionally, determining whether complete elimination or threshold-level exposure to allergens through the maternal diet is more beneficial for promoting oral tolerance in the infant is crucial. These findings would directly inform evidence-based clinical guidelines and help prevent overly restrictive practices. Additionally, it remains unknown whether eliminating an allergen from the maternal diet or reducing it to a certain threshold is more effective in promoting tolerance development in the infant, underscoring the need for targeted studies on this issue. Understanding these aspects can guide practical recommendations that avoid unnecessary restrictions while ensuring symptom resolution. The nutritional impact of prolonged elimination diets on mothers, including effects on nutrient status, bone health, and gut microbiota, warrants long-term study, along with the exploration of suitable alternatives to dairy that maintain probiotic intake. Given the infant gut microbiome’s crucial role in allergy development, examining how the maternal diet and breast milk composition influence this ecosystem is essential for developing preventive strategies. Embracing precision nutrition, future studies should utilize genetic, immunological, and microbiome profiling to provide tailored dietary advice that meets each infant’s needs while minimizing unnecessary restrictions. Research into caregiver education, professional counselling, and digital health tools is equally important to improve adherence to elimination diets, prevent nutritional deficiencies, and promote the overall well-being of both mother and child.

## Figures and Tables

**Table 1:** Manifestations of IgE-mediated reactions to cow’s milk proteins contained in breast milk [11,16,52].

Conditions	Notes
Cutaneous manifestations	Erythematous rashes, urticaria, and angioedema, typically involving the eyelids, face, and lips.
Respiratory symptoms	Rhinorrhea, sneezing, coughing, stridor, wheezing, and shortness of breath.
Gastrointestinal symptoms	Vomiting, crampy abdominal pain, and diarrhea.
Cardiovascular and neurological involvement	These symptoms usually accompany other systemic manifestations, such as respiratory or cutaneous reactions.
Anaphylaxis	It is the most severe form of IgE-mediated hypersensitivity reaction and involves multiple organ systems. It has a rapid onset and can be potentially life-threatening. Anaphylaxis in infants is rare and often presents with vomiting and hives, which can make early diagnosis difficult.

**Table 2:** Symptoms and times to assess the response to the elimination diet and to oral food challenge.

Clinical Presentation	Response to Elimination Diet	Response to Oral Food Challenge
IgE-mediated symptoms (anaphylaxis, urticaria, angioedema, gastrointestinal, cardiovascular, and respiratory symptoms)	2–3 days	Immediate, 2 h
Food protein-induced enterocolitis syndrome	2 days in acute form, 3–7 days in chronic form	1–4 h, typically 2 h
Food protein-induced proctocolitis	2–3 days	1–3 days
Food protein-induced enteropathy	1–4 weeks	40–72 h
Atopic dermatitis	15 days	4 days
